# A huge uterine leiomyoma prolapsed through the vagina: image in medicine

**DOI:** 10.1093/omcr/omac036

**Published:** 2022-04-19

**Authors:** Yassine Bouhtouri, Hamza Messaoudi, Ivaldino Venanvio Nabalim, Moulay Abdelah Baba Habib, Moulay Mehdi El Hassani, Jaouad Kouach

**Affiliations:** Department of Gynecology and Obstetrics, Military Hospital of Instruction Mohamed V Rabat, Rabat, Morocco; Department of Gynecology and Obstetrics, Military Hospital of Instruction Mohamed V Rabat, Rabat, Morocco; Department of Gynecology and Obstetrics, Military Hospital of Instruction Mohamed V Rabat, Rabat, Morocco; Department of Gynecology and Obstetrics, Military Hospital of Instruction Mohamed V Rabat, Rabat, Morocco; Department of Gynecology and Obstetrics, Military Hospital of Instruction Mohamed V Rabat, Rabat, Morocco; Department of Gynecology and Obstetrics, Military Hospital of Instruction Mohamed V Rabat, Rabat, Morocco

**Figure 1:**
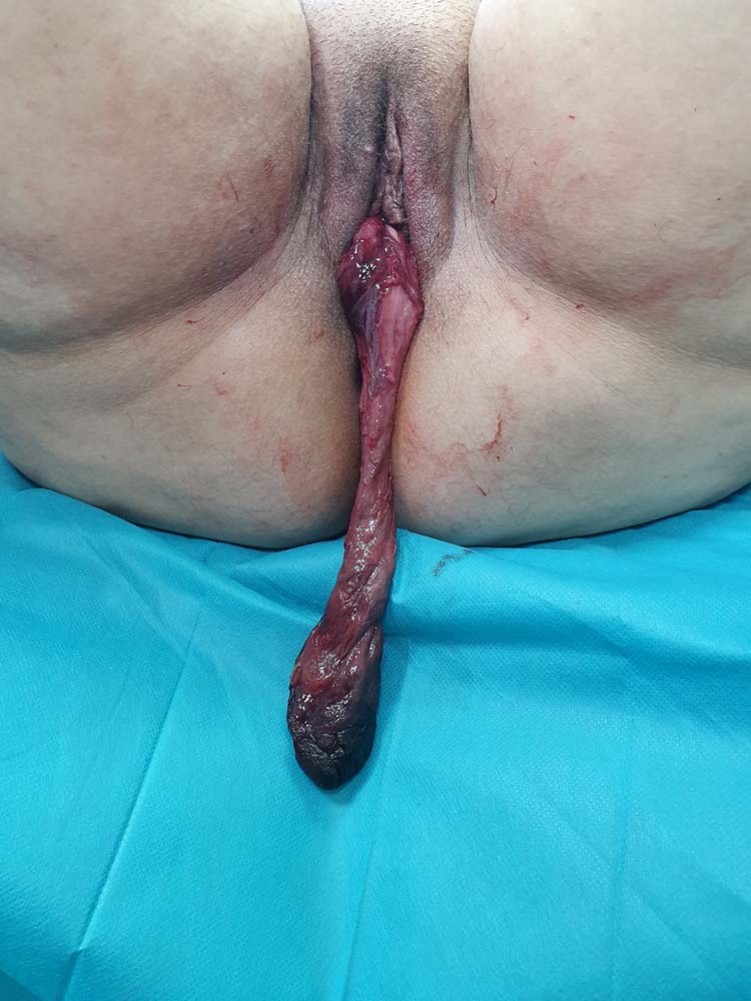
A huge uterine leiomyoma measuring 25 cm in length prolapsed through the vagina.

Leiomyomas are very common benign tumors of the uterus. They affect 20% of 30-year-old women and 50% of 50-year-old women, their frequency increases in case of smoking and nulliparity and they affect black women three or four times more often [1]. They are classified according to their location in the uterus according to Munro’s FIGO classification in 2011: subserous, intramural and submucosal. In some cases, submucosal pedunculated myomas dilate the cervix and protrude into the vagina [2]. We report the case of a patient; 42 years old, mother of three children, with no particular pathological history, who consulted our institution for a mass protruding through the vagina with the notion of menometrorrhagia evolving for 4 months. The clinical examination revealed a patient in good general condition. Gynecologically, a necrotic mass protruded through the vagina, measuring 25 cm in length (Fig. 1). Pelvic ultrasound was in favor of a type 0 uterine myoma according to the FIGO classification. The patient underwent vaginal myomectomy. The anatomopathological study confirmed the diagnosis of necrotic leiomyoma. The postoperative evolution was simple for the patient.
